# Design and Validation of a Questionnaire on Communicating Bad News in Nursing: A Pilot Study

**DOI:** 10.3390/ijerph17020457

**Published:** 2020-01-10

**Authors:** Manuel González-Cabrera, Ana Raquel Ortega-Martínez, Juan Miguel Martínez-Galiano, Antonio Hernández-Martínez, Laura Parra-Anguita, Antonio Frías-Osuna

**Affiliations:** 1 Department of Emergency and Critical Care, San Agustín University Hospital, 23700 Linares, Spain; 2 Nursing department, Faculty of Health Sciences, University of Jaen, 23071 Jaen, Spain; jgaliano@ujaen.es (J.M.M.-G.); lparra@ujaen.es (L.P.-A.); afrias@ujaen.es (A.F.-O.); 3 Department of Psychology, Faculty of Humanities and Education Sciences, University of Jaen, 23071 Jaen, Spain; arortega@ujaen.es; 4 Consortium for Biomedical Research in Epidemiology and Public Health (CIBERESP), 28029 Madrid, Spain; 5 Nursing department, Faculty of Nursing, University of Castilla la Mancha, 13071 Ciudad Real, Spain; antomatron@gmail.com

**Keywords:** communicating bad news, questionnaire, nursing, validation

## Abstract

Communicating bad news (CBN) is a fundamental skill in nursing; nevertheless, few instruments exist for its evaluation. This study presents a questionnaire designed to measure nurses’ knowledge and ability of CBN, as well as the analysis of its psychometric properties. Based on a literature search, the initial dimensions of CBN were identified to construct the questionnaires’ items, which were evaluated by experts for the validity of the items’ contents. Construct validity and reliability of the resulting questionnaire was carried out in a sample of 71 nurses of an Andalusian university hospital. A questionnaire with 25 items was constructed with a high internal consistency (Cronbach’s alpha 0.816). The content validity was evaluated via a literature review and additionally by the assessment of seven experts. The Kaiser-Meyer-Olkin test (KMO) obtained a score of 0.683, and the Bartlett test of sphericity a value of *p* < 0.001. The principal component analysis supported a construct of four dimensions. This questionnaire was found to be a valid and reliable instrument with a high internal consistency for the evaluation of CBN knowledge and skills of nursing professionals.

## 1. Introduction

Communication with a patient and their family is a basic and transcendental component of healthcare [1]. Frequently there are situations in which healthcare professionals, and therefore nursing professionals, are faced with the difficult task of communicating bad news (CBN) [2]. These situations arise during the process of care and disease evolution and can cause significant emotional distress—including anxiety, anger, etc.—in both the patient or family and healthcare professionals [3,4].

From the healthcare professional’s point of view, the most accepted concept of “bad news” (BN) is that by Buckman, who defines it as “that which will seriously or adversely change a patients perspectives of the future” [5,6]. CBN can result in difficult, aggressive, or emotionally unstable situations; therefore, it is vital that healthcare professionals develop and/or improve the knowledge, attitudes, and skills needed for this difficult task [1,3,4,7,8,9,10]. The importance of CBN is reflected in the fact that when patients and families receive BN, they find it hard to forget where, when and how the BN was communicated [4,9], highlighting the need for the professional to have excellent proficiency in this skill. Furthermore, although the content of the BN itself is inevitable and important, it is possible to mitigate its impact by improving communication skills and the place and manner of transmitting that content influences its impact.

The majority of practical clinical guidelines on CBN are aimed at medical doctors and, in general, the oncological field [3,11,12,13]. Nevertheless, nurses, who are faced with the difficult and frequent task of CBN in their day-to-day work [7,10,11], do not have these specific type of tools that help, orientate, and evaluate the process of CBN [14]. Various studies highlight these existing gaps in CBN knowledge, attitude and skills, justifying the need to create an instrument that evaluates how BN is communicated and that also identifies the need for continuing education in this area [1,3,4,8,9,10]. This is further justified when considering that the lack of these skills have important consequences for both the patients and their families, as well as for the healthcare professionals themselves [4,10,15,16,17].

Only two questionnaires exist that are specifically focused on CBN, and these are aimed at medical doctors that speak English or German. These questionnaires are the “Breaking Bad News Assessment Scale” (BAS) [18], and the questionnaire “Bad News Consultation Assessment Scale” (ACBS) [19]. There are also protocols that propose basic methodologies for CBN such as: Buckman [20], Advance preparation- Build a therapeutic environment/relationship-Commnunicat well-Deal with patient and family reactions- Encourage and validate emotions protocol (ABCDE protocol) [21], Background-Rapport-Explore-Announce-Kindling-Summarize protocol (BREAKS protocol) [22], and the ABCDE protocol adapted for nursing (ABCDEE); however, training in CBN for nursing professionals is scarce, self-directed, unstructured, and generalist [21]. No validated instrument exists to assess CBN skills and knowledge [1,4,7,8,9,10,23]. For this reason, we aimed to design a valid and reliable tool to evaluate the knowledge and skills in communicating bad news in nursing professionals.

## 2. Materials and Methods

### 2.1. Design

This study was a cross-sectional observational validation of a questionnaire. Ethical approval was given for this study by the Ethics Committee of Jaen (protocol number MGC17).

The first step in the design and construction of this questionnaire was a scientific literature review using the databases PubMed, LILACS, CINAHL, MEDLINE, and SCIELO. The following keywords were used: communicating bad news; questionnaire; nursing; validation. These keywords were combined with the adequate operators in each database.

Based on the defined construct, together with the protocols recommended by Buckman and Baile Setting-Perception-Invitation-Knowledge-Empathy-Strategy-Summary(SPIKES) [20], Rambow and McPhec (ABCDE) [24], and the adapted version for nursing by Villa López (ABCDEE) [25], six initial dimensions were established: (1) Preparation (preparation of the environment), (2) Perception (determine what is already known), (3) Invitation (establish what they want to know), (4) Knowledge (communicate correctly), (5) Empathy (process of active listening), and (6) Strategy (Establish a therapeutic plan or care plan).

In agreement with all the above, an initial questionnaire was constructed that comprised of 31 items: 7 related to Preparation, 3 with Perception, 4 with Invitation, 5 with Knowledge, 7 with Empathy, and 5 with Strategy.

This initial questionnaire was evaluated by seven experts chosen for their knowledge, diversity of ideas regarding the study topic, and commitment to collaboration. These experts had an adequate level of training in their field of work and in the communication of BN (basic and specialized), as well as clinical experience in different services in which it is customary to communicate BN (emergency, intensive care, palliative care, etc.). In addition, two profiles that had experience in managing any of these services were included. Two experts in validation and design of questionnaires were also part of the team. In accordance with the Delphi method (anonymity of the experts, repetitiveness, and controlled feedback), they were provided with a 5-point Likert type scale for the evaluation of the items, where 1 was the maximum value (maximum agreement) and 5 the minimum value (minimum agreement), assessing four aspects: wording, comprehension, pertinence, and general evaluation.

### 2.2. Selection of Study Participants

A convenience sample was selected for the pilot study comprising of 71 registered nurses from the critical care and emergency departments. The inclusion criteria were as follows: be working at the time of the study’s data collection and have at least one year of experience in the departments of critical care and emergency.

The potential participants for this study were identified using data provided by the hospital’s central management. Each participant was contacted, the objective of the study was explained, and the corresponding informed consent obtained. The questionnaire (self-administered) was provided to each participant at their work in the presence of one of the researchers. The possibility of loss to follow up during the study was minimized by establishing in person individual contact with each of the selected professionals to give them the questionnaire. The response rate of the participants was 86%.

### 2.3. Statistical Analysis

To determine the degree of agreement between the experts in the earlier selection of the items, a descriptive analysis of the data was conducted using the calculation of the median and interquartile range. Items with a median equal to or higher than 3 were excluded.

The reliability of the questionnaire was determined via analysis of its internal consistency using the Cronbach’s α coefficient. In order to discover the underlying structure of the data, how they behave, and how many factors they determine, a principal component analysis (PCA) was conducted (KMO and Bartlett’s test of sphericity). All analyses were performed using SPSS v24.0 statistics package (SPSS Inc., Chicago, IL, USA).

## 3. Results

A total of 71 nurses participated in this pilot study: 27 men (38%) and 44 women (62%). In terms of workplace, 45 nurses worked in the emergency department and 26 worked in critical care. Only 3% worked the day shift (7 h between 8 am and 10 pm), while 13% worked on-call shifts (17 h), and 84% worked on a duty rota (7 to 10 h).

In terms of content validity, the analysis of the decisions made by the experts showed a high degree of agreement in the adequacy of the items, with only three items excluded due to median calculations higher than two. The resulting initial version of the questionnaire included 28 items. The eliminated items were numbers 9, 24, and 25 (Table 1). The questionnaire was evaluated again by the seven experts, and the adequacy of the items was agreed on, with a median score for each between one and two points (maximum agreement).

With regards to the reliability of the questionnaire, Cronbach’s α was used to assess the degree of internal consistency and was calculated as 0.778.

To determine the validity of the questionnaire’s construct a PCA was conducted (32). The KMO test and Bartlett’s test of sphericity were calculated obtaining a value of *p* = 0.610 and a value of *p* < 0.01, respectively. Therefore, it was considered appropriate to conduct a factor analysis with all the data. Eleven components were obtained with eigenvalues greater than one that explained 71.60% of the questionnaire’s variance (Table 2).

When testing the measure of sampling adequacy, item 15 was observed to have a very low value (0.198); therefore, the analysis was conducted again excluding this item. In the new analysis a Cronbach’s alpha value of 0.788 was obtained. Items seven and 16 were shown to be prejudicial for consistency, and consequently a final PCA was conducted without these items. A KMO value of 0.683 was obtained and a Bartlett’s test of sphericity *p* < 0.001, therefore a factor analysis was conducted with all the data. This resulted in four components with eigenvalues greater than one that explained 43.20% of the variance of the questionnaire (Table 3). When verifying the measure of sampling adequacy for each item, we did not find the need to eliminate any further items. In the resulting component matrix, the weights for each item in the four components were tested, and to facilitate their interpretation a varimax rotation was conducted (Table 4). Once items seven, 15, and 16 were eliminated from the final questionnaire (25 items) (Table 5), a Cronbach’s alpha of 0.816 was obtained showing a high internal consistency.

## 4. Discussion

The CBN questionnaire for nursing professionals designed in this study, was shown to be an instrument with an adequate guarantee of reliability, content validity, and construct validity. The Cronbach’s α estimate was 0.816, showing good internal consistency and therefore reliability.

The lack of similar questionnaires prevents comparison with our results. Nevertheless, if we compare with other validated questionnaires on general communication, we find values similar to the value obtained in the GHATA-ENFERMERÍA questionnaire [26] with a value of 0.843, higher than in the GHATA-ESP questionnaire [27] (0.6121) and the GHATA-RES questionnaire [28] (0.76), while lower than that obtained in the CICCA scale [0.957] [29].

Comparing our questionnaire to the multi-dimensional theory proposed by the CBN protocols [24,25,28,29], which denote the existence of six dimensions, as well as the three axes that are put forward by existing communication questionnaires [26,27,28,29], we concluded that only four components were obtained in our study owing to the PCA. These four components explained 40.32% of the variance; hence our conclusion that the CBN questionnaire for nursing is established with a construct of four dimensions. If we compare the dimensions considered in our study with the dimensions of other studies, four versus six, it should be taken into account that these studies refer to BN communication protocols, not to questionnaires which was the purpose of our study. The establishment of four dimensions rather than six is justified in the results by the weight of each items in the four main factors. 

Given the absence of specific questionnaires on CBN in nursing and existence of questionnaires on only communication in general, this questionnaire occupies this empty space and is a strength of the study. Furthermore, in contrast to the questionnaires in guidelines using recordings of interviews between nurses and patients, this questionnaire is more widely usable and does not limit the number of professionals and situations that can benefit from its use. The field or department can be broadened to any type of context in which CBN is a daily reality for nurses, such as emergency services, intensive care units, hospitalization units, homes, consultations, etc.

In terms of limitations, only 40.32% of the total variance was explained, however, given that the present study is a pilot study, we would expect that a larger number of responses would resolve this deficiency in the model obtained and improve its relevance [30,31]. Given that a convenience sample was used, problems of non-representativity exist as well as attenuation due to the limitation of the range [32]. The Hawthorne effect was mitigated by anonymity and the voluntary nature of questionnaire completion; once participation had been accepted, the completion of the questionnaire was carried out by an anonymous nursing professional without any identifying data and later entered into a base of anonymized data for analysis. It would have been interesting to carry out the test–retest reliability, but the work overload of the participants, the transfer of units of some, and other circumstances made it difficult for this to be carried out. Hence, it is a limitation to take into account, although we believe that its influence on the results would be minimal.

This pilot study adds encouraging results and opens the door to continuing progress in this line of research. Furthermore, this tool can be regard as a very useful instrument and as a checklist among professionals that communicate BN to ensure they approach each situation in the correct manner. Just as it could be used in the future to understand and assess the way in which professionals communicate BN, and based on the deficiencies observed, develop an education and training plan for professionals.

## 5. Conclusions

The instrument created is valid and reliable for the evaluation of CBN by nursing staff and is able to detect possible deficiencies in knowledge and skills in communicating bad news. This resource will provide a useful, reliable and valid tool of great interest and use in the field of nursing where giving BN (about care, disease progression, etc.) is common, and it will be useful for each professional to self-check their ability to carry out the communication of BN and detect areas of improvement, and thus, communicating the BN in the best manner possible, the impact of its delivery can be minimized. Future lines of work will determine the validity of this tool among professionals from other disciplines and study the intraobserver reliability.

## Figures and Tables

**Table 1:** Evaluation of the items by the experts.

Items	Wording	Comprehension	Relevance	Global Assessment
	Median (IQR)	Median (IQR)	Median (IQR)	Median (IQR)
Item 1	1.5 (1)	1.0 (1)	1.0 (1)	1.0 (1)
Item 2	1.0 (1)	1.0 (1)	1.0 (1)	1.0 (1)
Item 3	2.0 (2)	2.0 (2)	2.0 (3)	2.0 (2)
Item 4	1.0 (1)	1.0 (1)	1.0 (1)	1.0 (1)
Item 5	1.0 (0)	1.0 (0)	1.0 (0)	1.0 (1)
Item 6	1.0 (1)	1.0 (0)	1.0 (1)	1.0 (0)
Item 7	2.0 (2)	1.5 (1)	1.0 (0)	1.5 (2)
Item 8	2.0 (2)	2.0 (2)	1.0 (2)	2.0 (2)
Item 9	3.0 (1)	3.0 (1)	1.5 (1)	2.0 (2)
Item 10	2.5 (2)	2.0 (2)	2.0 (3)	2.5 (2)
Item 11	1.0 (1)	1.0 (1)	2.5 (3)	1.5 (3)
Item 12	2.0 (3)	1.5 (1)	1.0 (2)	2.0 (2)
Item 13	2.0 (3)	1.5 (2)	2.0 (2)	1.5 (2)
Item 14	1.5 (2)	1.0 (1)	1.0 (1)	1.0 (1)
Item 15	2.5 (2)	2.0 (1)	1.0 (0)	1.5 (1)
Item 16	2.0 (2)	1.5 (1)	2.0 (2)	1.5 (2)
Item 17	1.5 (1)	1.0 (1)	1.5 (3)	1.0 (1)
Item 18	2.0 (2)	2.0 (1)	1.5 (2)	2.0 (1)
Item 19	1.5 (2)	1.5 (1)	1.0 (1)	1.5 (1)
Item 20	1.5 (2)	1.0 (1)	1.0 (2)	1.0 (1)
Item 21	2.0 (1)	1.5 (2)	1.5 (2)	1.5 (1)
Item 22	2.0 (1)	2.0 (0)	2.0 (1)	2.0 (1)
Item 23	1.5 (1)	2.0 (2)	1.0 (1)	1.0 (1)
Item 24	2.0 (2)	3.0 (1)	2.0 (2)	2.0 (2)
Item 25	2.5 (3)	2.5 (3)	2.0 (2)	2.5 (2)
Item 26	1.5 (1)	1.5 (2)	1.0 (3)	1.0 (2)
Item 27	2.0 (1)	2.0 (2)	1.0 (2)	1.5 (1)
Item 28	1.0 (1)	2.0 (2)	1.0 (2)	1.5 (2)
Item 29	1.5 (2)	1.5 (1)	1.5 (1)	1.5 (1)
Item 30	2.0 (3)	2.0 (2)	1.0 (1)	1.0 (1)
Item 31	1.0 (0)	1.0 (0)	1.0 (1)	1.0 (1)

IQR: Interquartile range. Wording (1 point: Very well written; 2 points: Well written; 3 points: Acceptable; 4 points: Poorly written; 5 points: Very poorly written). Comprehension (1 point: Good; 2 points: Sufficient; 3 points: Ok; 4 points: Poor; 5 points: Very poor). Relevance (1 point: Very relevant; 2 points: Quite relevant; 3 points: Relevant; 4 points: Not very relevant; 5 points: Not relevant). Global assessment (1 point: Very good; 2 points: Good; 3 points: Ok; 4 points: Poor; 5 points: Very poor).

**Table 2:** Total explained variance.

Component	Initial Eigenvalues			Extraction Sums of Squared Loadings		
	Total	% of Variance	Cumulative %	Total	% of Variance	Cumulative%
1	5.147	18.381	18.381	5.147	18.381	18.381
2	2.384	8.516	26.897	2.384	8.516	26.897
3	1.835	6.554	33.452	1.835	6.554	33.452
4	1.774	6.337	39.788	1.774	6.337	39.788
5	1.529	5.461	45.250	1.529	5.461	45.250
6	1.401	5.002	50.252	1.401	5.002	50.252
7	1.341	4.790	55.042	1.341	4.790	55.042
8	1.273	4.546	59.588	1.273	4.546	59.588
9	1.222	4.364	63.953	1.222	4.364	63.953
10	1.110	3.963	67.916	1.110	3.963	67.916
11	1.034	3.692	71.608	1.034	3.692	71.608
12	0.887	3.169	74.777			
13	0.815	2.909	77.686			
14	0.736	2.627	80.313			
15	0.705	2.518	82.832			
16	0.657	2.347	85.179			
17	0.605	2.160	87.339			
18	0.513	1.830	89.169			
19	0.435	1.553	90.723			
20	0.404	1.445	92.167			
21	0.370	1.321	93.488			
22	0.336	1.199	94.687			
23	0.320	1.143	95.830			
24	0.284	1.014	96.844			
25	0.274	0.980	97.823			
26	0.252	0.900	98.723			
27	0.226	0.807	99.530			
28	0.132	0.470	100.000			

Extraction method: Principal Components Analysis.

**Table 3:** Total explained variance.

Component	Initial Eigenvalues			Extraction Sums of Squared Loadings		
	Total	% of Variance	Cumulative %	Total	% of Variance	Cumulative %
1	5.088	20.354	20.354	5.088	20.354	20.354
2	2.322	9.289	29.643	2.322	9.289	29.643
3	1.781	7.123	36.766	1.781	7.123	36.766
4	1.610	6.439	43.205	1.610	6.439	43.205
5	1.472	5.886	49.091			
6	1.331	5.323	54.414			
7	1.241	4.966	59.379			
8	1.120	4.482	63.861			
9	1.058	4.233	68.094			
10	0.973	3.892	71.986			
11	0.830	3.320	75.306			
12	0.770	3.081	78.387			
13	0.721	2.884	81.272			
14	0.657	2.626	83.898			
15	0.612	2.447	86.345			
16	0.511	2.043	88.389			
17	0.443	1.774	90.162			
18	0.413	1.651	91.813			
19	0.402	1.609	93.422			
20	0.371	1.483	94.906			
21	0.289	1.158	96.064			
22	0.285	1.139	97.202			
23	0.274	1.097	98.299			
24	0.230	0.921	99.220			
25	0.195	0.780	100.000			

Extraction method: Principal Components Analysis.

**Table 4:** Component rotation matrix

	Component			
	1	2	3	4
Item1	0.675	−0.011	−0.003	−0.050
Item2	0.645	−0.264	0.105	0.058
Item3	0.414	−0.508	−0.053	0.263
Item4	−0.070	−0.076	0.612	0.160
Item5	0.051	0.212	0.442	0.302
Item6	0.090	0.036	0.099	0.647
Item8	0.156	−0.206	0.663	0.279
Item9	0.473	−0.044	−0.076	0.418
Item10	0.534	−0.076	0.141	0.313
Item11	0.491	0.243	−0.101	0.478
Item12	0.394	0.313	0.431	−0.196
Item13	0.253	0.562	0.456	0.070
Item14	0.034	0.668	0.113	0.114
Item17	0.678	0.137	−0.119	−0.009
Item18	0.453	0.138	0.268	−0.022
Item19	0.112	0.441	−0.071	−0.022
Item20	0.055	0.413	0.121	0.242
Item21	0.079	0.059	0.115	0.569
Item22	−0.020	0.017	0.048	0.422
Item23	0.125	0.230	0.712	−0.074
Item24	0.294	0.504	−0.116	0.262
Item25	0.548	0.337	0.223	0.003
Item26	0.644	0.316	0.163	0.006
Item27	0.508	0.234	0.100	0.135
Item28	−0.133	0.437	0.166	0.563

Extraction method: Principal components analysis. Rotation method: Varimax with Kaiser normalization a. Rotation converged in eight iterations.

**Table 5:** Nursing questionnaire on communicating bad news.

		Never 1	Sometimes 2	Always 3	NA 4
**1º**	Do you choose a quiet and private place beforehand to communicate bad news?	1	2	3	4
**2º**	Do you ensure that there will be no foreseeable interruption occurring (phone, consult by a colleague, etc.)?	1	2	3	4
**3ª**	Do you plan the duration?	1	2	3	4
**4º**	Do you introduce yourself to the patient first?	1	2	3	
**5º**	Do you call the patient by their name?	1	2	3	4
**6º**	Do you look at the patients face or in the eyes while you talk or listen?	1	2	3	4
**7º**	Before starting the conversation, do you find out what the patient already knows about the news that you are going to communicate?	1	2	3	4
**8º**	To find out what the patient knows and how much they want to know, do you use questions such as: Before I talk, do you want to tell me anything or ask me something?	1	2	3	4
**9º**	Before communicating bad news, do you find out in what way the news may affect the patient’s personal, social or work life?	1	2	3	4
**10ª**	In the event that the patient is unsure they wish to be informed, do you give the patient time to consider it?	1	2	3	4
**11º**	Do you tend to facilitate dialog with the patient or let them vent/blow off steam talking?	1	2	3	4
**12º**	Do you keep in the mind the opinion of the patient?	1	2	3	4
**13ª**	Do you use appropriate language to allow the patient to digest the bad news?	1	2	3	4
**14º**	Do you communicate the bad news sequentially and in an organized manner, not giving more information until you are sure that the information already given has been digested?	1	2	3	4
**15º**	Do you ask a question to find out how the patient is feeling?	1	2	3	4
**16º**	In terms of the feelings, fears and worries of the patient, do you verbally express your awareness or responsiveness?	1	2	3	4
**17ª**	When the patient’s response is anxiety, fear, sadness or aggression, do you maintain an attitude of active listening?	1	2	3	4
**18º**	Do you show support and understanding non-verbally?	1	2	3	4
**19ª**	When you communicate bad news, do you present yourself assertively, expressing your thoughts confidently?	1	2	3	4
**20º**	If a disagreement with the patient exists, do you wait for their input and find a solution to the problem?	1	2	3	4
**21º**	Do you observe the emotions that have emerged in the patient following the communication of bad news?	1	2	3	4
**22º**	Do you ensure that at the end of the conversation the patient has no further doubts or questions?	1	2	3	4
**23º**	Do you establish, if necessary, a care plan together with the patient to address the new situation?	1	2	3	4
**24ª**	Do you explore the possible occurrence of challenging situations after the communication of bad news and establish a strategy for future action?	1	2	3	4
**25ª**	Do you farewell the patient at the end of the conversation?	1	2	3	4

Source: Our own development. Translated version for publication. Source: Own design.
